# Red blood cell acetylcholinesterase activity among healthy dwellers of an agrarian region in Sri Lanka: a descriptive cross-sectional study

**DOI:** 10.1186/s12199-018-0717-0

**Published:** 2018-06-21

**Authors:** Devarajan Rathish, Indika Senavirathna, Channa Jayasumana, Suneth Agampodi

**Affiliations:** 1 Department of Pharmacology, Faculty of Medicine and Allied Sciences, Rajarata University of Sri Lanka, Saliyapura, Sri Lanka; 2 Department of Biochemistry, Faculty of Medicine and Allied Sciences, Rajarata University of Sri Lanka, Saliyapura, Sri Lanka; 3 Department of Community Medicine, Faculty of Medicine and Allied Sciences, Rajarata University of Sri Lanka, Saliyapura, Sri Lanka

**Keywords:** Acetylcholinesterase activity, Organophosphate, Carbamate, Healthy volunteers

## Abstract

**Background:**

Assessment of acetylcholinesterase-inhibitor insecticide (AChEII) toxicity depends on the measurement of red blood cell acetylcholinesterase (RBC-AChE) activity. Its interpretation requires baseline values which is lacking in scientific literature. We aim to find the measures of central tendency and variation for RBC-AChE activity among dwellers of Anuradhapura, where the use and abuse of AChEIIs were rampant for the last few decades.

**Methods:**

A descriptive cross-sectional study with a community-based sampling for 100 healthy non-farmers (male:female = 1:1) was done using pre-determined selection criteria. Duplicate measurements of RBC-AChE activity were performed according to the modified Ellman procedure. Pearson’s correlation and regression analysis were sort for RBC-AChE activity against its possible determinants.

**Results:**

RBC-AChE activity had a mean of 449.8 (SD 74.2) mU/μM Hb with a statistical power of 0.847. It was similar to values of “healthy controls” from previous Sri Lankan toxicological studies but was low against international reference value [586.1 (SD 65.1) mU/μM Hb]. None of the possible determinants showed a significant strength of relationship with RBC-AChE activity.

**Conclusion:**

The baseline RBC-AChE activity among people of Anuradhapura is low in comparison with international reference values. This arises a need to find a causative mechanism.

**Electronic supplementary material:**

The online version of this article (10.1186/s12199-018-0717-0) contains supplementary material, which is available to authorized users.

## Background

Acetylcholinesterase (AChE) hydrolyses acetylcholine (ACh) at the cholinergic pathways of central and peripheral nervous systems. This leads to a resting state which is essential for further uninterrupted neurotransmission [1]. AChE inhibitors lead to accumulation of ACh and subsequent overstimulation of cholinergic receptors which disrupts neurotransmission. These inhibitors are used as therapeutic agents in myasthenia gravis, Alzheimer’s disease, Parkinson’s disease etc. [2]. However, toxic agents like organophosphates (OP) and carbamates are used against pests in farming [3]. Ethanol is another agent which could result in the inhibition of AChE activity [4].

AChE inhibitor-insecticides (AChEIIs) lead to acute or chronic poisoning in human. Self-harm and accidental exposures bring about acute poisoning, whereas chronic poisoning occurs among certain groups of workers like farmers. Red blood cell (RBC) AChE activity is used as a marker of toxicity, as it correlates better with the central nervous system AChE [5]. Globally, pesticides lead to 250,000 deaths per year, out of 3 million episodes of poisoning [6]. OP poisoning has a case fatality rate of 5–20% in Asia, whereas it is 5.8% for Anuradhapura [7, 8].

Assessing acute or occupational toxicity to AChE inhibitors requires an understanding of the baseline RBC-AChE activity for the geographical area, which is lacking in scientific literature. This study aims at finding the measures of central tendency, variation for RBC-AChE activity and its relationship with the possible determinants, among healthy non-farmers of Anuradhapura.

## Methods

A descriptive cross-sectional study using a community-based convenience sample of 100 healthy non-farmer volunteers (male:female = 1:1) was done at Anuradhapura during the month of September 2017. Anuradhapura is a district of North Central Province, Sri Lanka, where the majority belong to the rural sector (94.1%) [9] and agriculture (55%) is its main employment [10]. In 2014, the Sri Lankan government implemented a ban on three AChEIIs (chlorpyrifos, carbofuran, carbaryl) in Anuradhapura, as a response to increased toxicity [11]. Further, two other AChEIIs (dimethoate, fenthion) were banned from the country during the same year.

Inclusion criteria of the study were Buddhists aged ≥ 18 years and ≤ 65 years, estimated glomerular filtration rate (eGFR) ≥ 60 ml/min/1.73m^2^ according to CKD-EPI equation and permanent residence of Anuradhapura for ≥ 5 years. Exclusion criteria were any acute illness, history of acute organophosphate/carbamate poisoning, history of farming, history of use of acetylcholinesterase inhibitor medications, history of renal failure, malignancy, immunosuppression, haemoglobinopathies or anaemia, every day smokers [12], heavy alcohol users [13] and pregnancy.

The study was carried out to obtain demographic data, anthropometric measurements, blood pressure measurement and blood samples for eGFR and RBC-AChE activity. Study description, obtaining written informed consent, data collection and relevant physical examination were done by the first author. All necessary measures were taken to preserve participant’s privacy and confidentiality. Duplicate measurements of RBC-AChE activity in whole blood per haemoglobin concentrate was performed at the Department of Biochemistry, Faculty of Medicine and Allied Sciences, Rajarata University of Sri Lanka, according to the modified Ellman procedure [14, 15]. Sigma-Aldrich reagents, USA [16] were used as recommended, and the quality control was done using the AChE-check-control (high/low) from Securetec Detektions-Systeme AG, Germany [17]. Spectrophotometric reading was done using Spectro 2000, Labomed, Inc., USA [18]. Wavelengths of 546 and 436 nm were used for the measurement of haemoglobin content and RBC-AChE activity respectively. The measurement of RBC-AChE activity was done at a pH of 7.4 and at a temperature of 37 °C. The concentration in solution of Ellman’s reagent, ethopropazine hydrochloride and acetylthiocholine iodide were 10, 6 and 28.4 mM respectively [14, 15]. Data was entered to a Microsoft Excel sheet (Additional file 1). Measures of central tendency and variation of RBC-AChE activity were described. Two-sample *T* test was performed to determine significant difference in the means of RBC-AChE activity between males and females (*p* < 0.05). Pearson’s correlation was sort for RBC-AChE activity against age, years residing at Anuradhapura, waist circumference, weight, height, body mass index (BMI), mean arterial pressure (MAP) and eGFR. Regression analysis was done to determine the strength of the relationship between RBC-AChE activity and its above-mentioned possible determinants.

## Results

Majority of the participants were from Nuwaragam Palata East divisional secretariat division (52%), employed (92%) and educated up to or above the advanced level of general certificate of education (88%). The means (SD) for possible determinants of RBC-AChE activity are shown in Table 1.

**Table 1:** Possible determinants of red blood cell acetylcholinesterase activity among healthy, non-farmers of Anuradhapura, 2017

Factors	Mean (SD)	Pearson’s correlation coefficient	Regression analysis (*P* value)
Age (years)	36.3 (10.2)	− 0.142642318	0.156
Years residing at Anuradhapura (years)	32.3 (13.7)	− 0.026661425	0.274
Waist circumference (cm)	84.6 (9.6)	− 0.125366133	0.569
Weight (kg)	63.9 (13.5)	− 0.142078498	0.173
Height (cm)	161.6 (9.3)	− 0.051650597	0.291
Body mass index (kgm^−2^)	24.4 (4.4)	− 0.124457504	0.275
Mean blood pressure (mmHg)	89.6 (7.7)	+ 0.036766139	0.194
Estimated glomerular filtration rate (ml/min/1.73m^2^)	107.9 (15.5)	+ 0.138656806	0.760

**Fig. 1:**
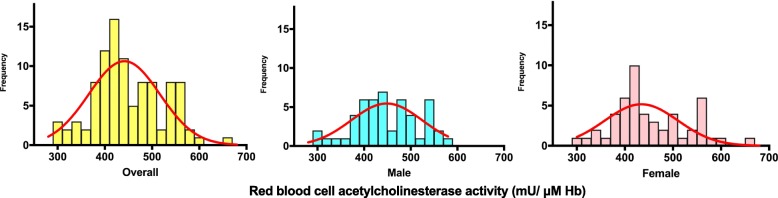
Distribution of acetylcholinesterase activity among healthy non-farmers of Anuradhapura, 2017

The RBC-AChE activity followed a near normal distribution (Fig. 1) and ranged from 290.4 to 669.1 mU/μM Hb (18.0 to 41.5 U/g Hb). Kurtosis and skewness for the distribution were − 0.1997 and + 0.3149 respectively. The standard error of kurtosis and skewness were 0.490 [√(24/n)] and 0.245 [√(6/n)] respectively. Therefore, absolute values of both kurtosis and skewness fall within ± 2 times of its standard errors, which indicate that the data are symmetric and showing near normality. The mean of RBC-AChE activity was 449.8 (SD 74.2) mU/μM Hb with a lower and upper 95% confidence interval of 435.1 and 464.5 respectively. The median was 435.2 mU/μM Hb with an interquartile range of 400.3 to 503.7. The statistical power was calculated as 0.847 using the statistical analysis system (SAS) university edition [19]. Power was calculated for a normal distribution and a one sample two-sided *T* test using the following parameters: study mean (449.8), mean from previous local literature (431.6) [17], standard deviation (60.4), sample size (100) and type I error (0.05).

**Fig. 2:**
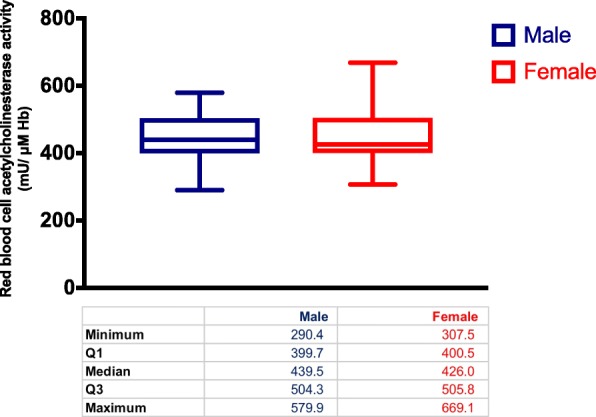
Red blood cell acetylcholinesterase activity by sex, among healthy non-farmers of Anuradhapura, 2017

**Fig. 3:**
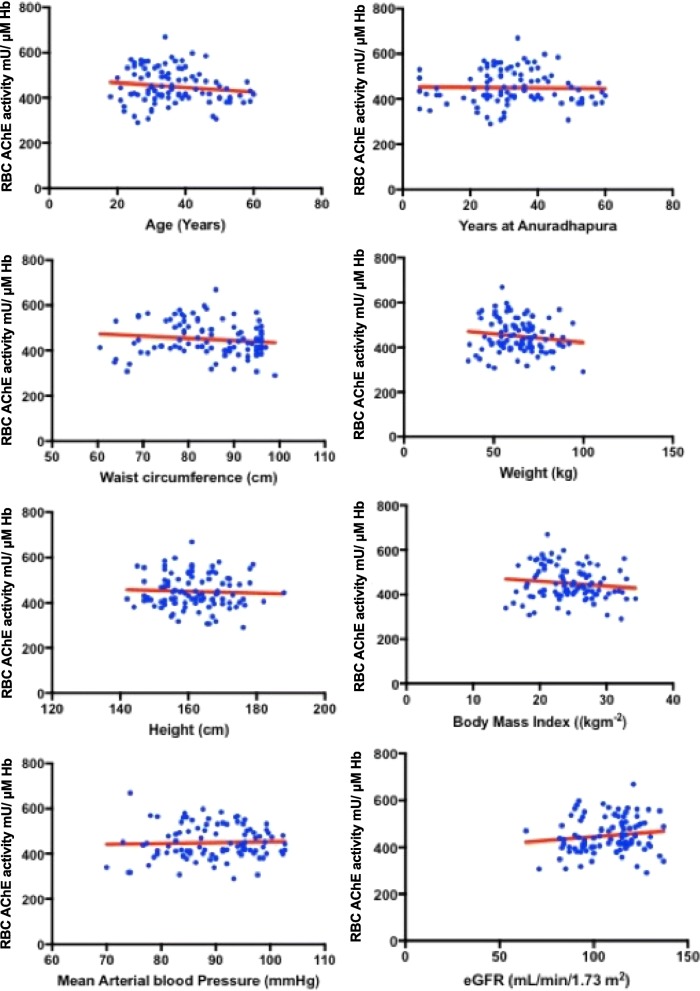
Scatter plot for RBC-AChE activity against its possible determinants among healthy non-farmers of Anuradhapura, 2017

There was no significant difference between the means of RBC-AChE activity for males (453.2 mU/μM Hb) and females (446.4 mU/μM Hb) (*p* = 0.649) (Fig. 2). The RBC-AChE activity showed a weak, negative correlation with age, waist circumference, weight and BMI. It showed a weak, positive correlation with eGFR (Table 1, Fig. 3). Regression analysis had an adjusted *R* square of 0.0079, showing poor overall regression accuracy. There was a high probability for the regression output to be by chance (significance *F* = 0.371). *P* values of all possible determinants failed to show any significance against RBC-AChE activity (Table 1). The distribution of the residuals was highly scattered too (Additional file 2).

## Discussion

Present study was conducted in Anuradhapura during an off-cultivation period (due to drought and lack of fertilisers). In-addition, it has been 3 years of ban on five hazardous AChE inhibitor-insecticides in Anuradhapura. A mean RBC-AChE activity of 449.8 (SD 74.2) mU/μM Hb was similar to activities found among “healthy-controls” of previous toxicological studies at the Southern Province of Sri Lanka. In 2002, RBC-AChE activity among 30 fishermen of the Southern Province decreased from 427.3 (SD 42.5) to 401.1 (SD 41.4) mU/μM Hb (*P* < 0.01) during cultivation [20]. In 2003, the decrease was 445.9 (SD 55.3) to 431.6 (SD 60.4) mU/μM Hb among 44 fishermen of the Southern Province [21]. However, “healthy controls” of a toxicological study done in Western Province (minimum cultivation) showed a higher activity (525.2 mU/μM Hb) among 50 fishermen [22]. These confirm a decrease in RBC-AChE activity during cultivation, even among the “healthy”.

Our findings as well as findings from Southern Province were lower than the reported data from other countries. Worek F et al. showed a RBC-AChE activity of 586.1 (SD 65.1) mU/μM Hb among ten native samples [15]. The manual of Test-mate-ChE suggests 544.5 (12.9) mU/μM Hb as the normal activity [23]. Indirect chronic exposure to AChEIIs could be an explanation for lower RBC-AChE activity among healthy non-farmers of an agrarian region. Using a modified Michel method, the largest similar study reported 0.74 ± 0.06 delta pH units/h as the normal range for RBC-AChE activity among 991 individuals of the USA [24]. However, comparison was difficult due to the lack of a conversion factor between the delta pH assay and the Ellman’s assay results [24].

RBC-AChE activity of the healthy non-farmers had a weak, negative correlation with waist circumference, weight and BMI. Inhibition of AChE at the incretin pathway has been implicated with diabetes mellitus [25]. A similar mechanism could be an explanation for the above observations. However, a meta-analysis has shown a weight reduction among dementia patients treated with AChE inhibitors [26]. Therefore, further studies focused on the association between RBC-AChE activity and anthropometric measurements are vital, especially among dwellers of agrarian regions.

This study lacks adequate comparison due to unavailability of previous similar local, regional or global data. However, it had produced valuable baseline RBC-AChE activity among healthy, non-farmers of an agrarian region which could be used in future toxicological studies related to AChE inhibitors. To the best of our knowledge, this is the largest study on RBC-AChE activity, among male and female volunteers, using Ellman’s method.

## Conclusion

The baseline mean RBC-AChE activity was low compared to international references. Further studies to find reasons for persistent low activity are essential. In-addition, RBC-AChE activity failed to show any significant strength of relationship with the possible determinants considered.

## Additional files

Additional file 1: RBC AChE activity among healthy non-farmers of Anuradhapura, 2017. It contains the data of the entire study. (XLS 49 kb)

Additional file 2: Regression analysis: RBC-AChE activity against its possible determinants among healthy non-farmers of Anuradhapura, 2017. It contains the regression analysis for RBC-AChE activity against its possible determinants. (PDF 404 kb)

## Abbreviations

ACh: Acetylcholine
AChE: Acetylcholinesterase
AChEIIs: Acetylcholinesterase inhibitor-insecticides
BMI: Body mass index
CKD-EPI: Chronic kidney disease epidemiology collaboration
eGFR: Estimated glomerular filtration rate
Hb: Haemoglobin
MAP: Mean arterial blood pressure
OP: Organophosphate
RBC: Red blood cell
SD: Standard deviation
SE: Standard error

## References

[1] Colovic MB, Krstic DZ, Lazarevic-Pasti TD, Bondzic AM, Vasic VM (2013). Acetylcholinesterase inhibitors: pharmacology and toxicology. Curr Neuropharmacol.

[2] Giacobini E (2004). Cholinesterase inhibitors: new roles and therapeutic alternatives. Pharmacol Res.

[3] Vale A, Lotti M (2015). Organophosphorus and carbamate insecticide poisoning. Handb Clin Neurol.

[4] Haboubi NA, Thurnham DI (1986). Effect of ethanol on erythrocyte acetylcholinesterase activity. Ann Clin Biochem.

[5] Katz KD. Organophosphate Toxicity Workup: Laboratory Studies, Imaging Studies, Electrocardiography. Medscape. 2015 [cited 2018 Feb 26]. Available from: http://emedicine.medscape.com/article/167726-workup

[6] McNab C. WHO | Pesticides are a leading suicide method: WHO Communications; 2006. [cited 2018 Feb 4]; Available from: http://www.who.int/mediacentre/news/notes/2006/np24/en/

[7] Thomas SHL, White J, Walker BR, Colledge NR, Ralston SH, Penman ID (2014). Organophosphorus insecticides and nerve agents. Davidson’s Principles and Practice of Medicine. 22nd ed.

[8] Senarathna L, Jayamanna SF, Kelly PJ, Buckley NA, Dibley MJ, Dawson AH (2012). Changing epidemiologic patterns of deliberate self poisoning in a rural district of Sri Lanka. BMC Public Health.

[9] Census of Population and Housing, Department of Census and Statistics, Ministry of Policy Planning and Economic Affairs, Sri Lanka. 2012 [cited 2018 Feb 4]. p. 52. Available from: http://www.statistics.gov.lk/PopHouSat/CPH2011/Pages/Activities/Reports/FinalReport/FinalReportE.pdf.

[10] Annual Bulletin, Sri Lanka Labour Force Survey, Department of Census and Statistics, Ministry of Finance and Planning. 2014 [cited 2018 Feb 4]. p. 2–3. Available from: http://www.statistics.gov.lk/samplesurvey/LFS_Annual Bulletin_2014-f.pdf.

[11] Gazette [Internet]. Department of Government Printing. 2018 [cited 2018 Feb 4]. Available from: http://documents.gov.lk/en/gazette.php

[12] Adult Tobacco Use Information. National Health Interview Survey. 2015 [cited 2017 Mar 10]. Available from: https://www.cdc.gov/nchs/nhis/tobacco/tobacco_glossary.htm

[13] Drinking Levels Defined [Internet]. National Institute on Alcohol Abuse and Alcoholism. 2016 [cited 2017 Mar 10]. Available from: https://www.niaaa.nih.gov/alcohol-health/overview-alcohol-consumption/moderate-binge-drinking

[14] Worek F. Standard operation procedure - Determination of cholinesterase status in whole blood and plasma. 4.1 ed. Institut für Pharmakologie und Toxikologie der Bundeswehr, München; 2013.

[15] Worek F, Mast U, Kiderlen D, Diepold C, Eyer P (1999). Improved determination of acetylcholinesterase activity in human whole blood. Clin Chim Acta.

[16] Sigma-Aldrich, analytical reagents and solvents [Internet]. Merck KGaA, Darmstadt, Germany. 2018 [cited 2018 Feb 8]. Available from: https://www.sigmaaldrich.com/analytical-chromatography/analytical-reagents.html

[17] AChE check Control high/low. Securetec Detektions-Systeme AG, Germany. 2018 [cited 2018 Feb 8]. Available from: https://www.securetec.net/sites/default/files/03_Produkte/ChECheck/Dateien/ache_control_acetylcholinesterase_flyer_70531_en_v01.pdf

[18] Spectro 2000. Labomed, Inc, USA. 2001 [cited 2018 Feb 8]. Available from: http://www.labomed.com/2000rs.htm

[19] SAS® University Edition [Internet]. SAS Institute Inc., USA. 2018 [cited 2018 Feb 8]. Available from: https://www.sas.com/en_us/software/university-edition.html

[20] Peiris-John RJ, Ruberu DK, Wickremasinghe AR, Smit LAM, Van der Hoek W (2002). Effects of occupational exposure to organophosphate pesticides on nerve and neuromuscular function. J Occup Environ Med.

[21] Smit LA (2003). Neurological symptoms among Sri Lankan farmers occupationally exposed to acetyl cholinesterase-inhibiting insecticides. Am J Indian Med.

[22] Peiris-John R, Wanigasuriya J, Wickremasinghe A, Dissanayake W, Hittarage A (2009). Exposure to acetylcholinesterase-inhibiting pesticides and chronic renal failure. Ceylon Med J.

[23] Test-mate ChE Cholinesterase Test System (Model 400) - Instruction Manual. [Internet]. EQM Research, Inc. Ohio; 2003 [cited 2018 Feb 4]. p. 18. Available from: http://www.eqmresearch.com/Manual-E.pdf

[24] Arrieta DE, McCurdy SA, Henderson JD, Lefkowitz LJ, Reitstetter R, Wilson BW. Normal range of human red blood cell acetylcholinesterase activity. Drug Chem Toxicol. 2009;32(3):182–5. 10.1080/01480540902863440 19538013

[25] Rathish D, Agampodi SB, Jayasumana MACS, Siribaddana SH (2016). From organophosphate poisoning to diabetes mellitus: the incretin effect. Med Hypotheses.

[26] Soysal P, Isik AT, Stubbs B, Solmi M, Volpe M, Luchini C (2016). Acetylcholinesterase inhibitors are associated with weight loss in older people with dementia: a systematic review and meta-analysis. J Neurol Neurosurg Psychiatry.

