# Supplementary material for: Red blood cell acetylcholinesterase activity among healthy dwellers of an agrarian region in Sri Lanka: a descriptive cross-sectional study
Source: Environ Health Prev Med. 2018 Jun 21;23:25. doi: 10.1186/s12199-018-0717-0 (PMC6013882; doi:10.1186/s12199-018-0717-0)
Supplement: Supplementary file 2 — Regression analysis: RBC-AChE activity against its possible determinants among healthy non-farmers of Anuradhapura, 2017. It contains the regression analysis for RBC-AChE activity against its possible determinants. (PDF 404 kb) [file 12199_2018_717_MOESM2_ESM.pdf]

Additional file – 2 Regression analysis: RBC-AChE activity against its possible determinants among healthy, non-farmers of Anuradhapura, 2017

Regression Statistics	
Multiple R	0.296827494
**R Square**	0.088106561
**Adjusted R Square**	0.007940105
Standard Error	73.85087652
Observations	100

ANOVA

	df	*SS*	*MS*	*F*	***Significance F***
Regression	8	47953.11892	5994.139865	1.0990452	0.37131779
Residual	91	496309.6287	5453.951963		
Total	99	544262.7476			

	Coefficients	*Standard Error*	*t Stat*	***P-value***	*Lower 95%*	*Upper 95%*	*Lower 95.0%*	*Upper 95.0%*
**Intercept**	-505.2842976	835.0744627	-0.60507694	0.5466338	-2164.05713	1153.4885	-2164.057	1153.48854
**Age**	-2.187090259	1.529994935	-1.42947549	0.1562917	-5.22623724	0.8520567	-5.226237	0.85205673
**Years @ Apura**	0.889597953	0.808586323	1.100189216	0.2741516	-0.71655945	2.4957554	-0.716559	2.49575535
**Waist C (cm)**	1.02951262	1.799820485	0.572008502	0.5687266	-2.54560967	4.6046349	-2.54561	4.60463491
**Weight**	-8.441149036	6.151101474	-1.37229878	0.1733436	-20.6595565	3.7772584	-20.65956	3.77725841
**Height**	5.431380834	5.115869277	1.061673108	0.2911936	-4.73066502	15.593427	-4.730665	15.5934267
**BMI**	17.50726594	15.94653525	1.097872714	0.2751565	-14.1685674	49.183099	-14.16857	49.1830993
**Mean BP**	1.45508666	1.111568628	1.309038977	0.1938171	-0.75290787	3.6630812	-0.752908	3.66308119
**eGFR**	0.214081251	0.698567747	0.306457393	0.7599565	-1.17353776	1.6017003	-1.173538	1.60170026

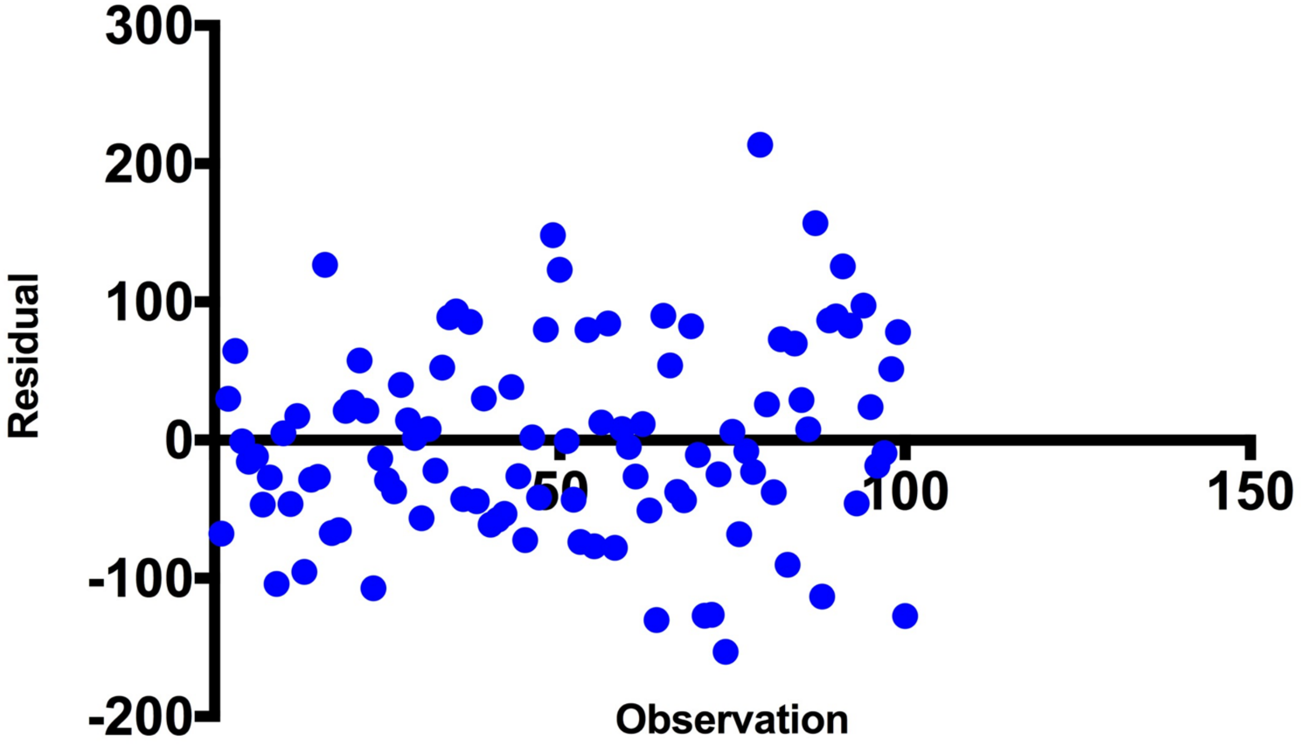

Distribution of Residuals
